# Case Report: Hemangioblastoma- Like Clear Cell Stromal Tumor of the Left Lower Lung

**DOI:** 10.3389/fmed.2022.836012

**Published:** 2022-04-18

**Authors:** Xiaowei Zhang, Bifei Huang, Hongquan Jiang, Hangping Wei

**Affiliations:** ^1^Department of Pathology, Affiliated Dongyang Hospital of Wenzhou Medical University, Dongyang, China; ^2^Department of Thoracic Surgery, Affiliated Dongyang Hospital of Wenzhou Medical University, Dongyang, China; ^3^Department of Medical Oncology, Affiliated Dongyang Hospital of Wenzhou Medical University, Dongyang, China

**Keywords:** *Yap1*, TFE3, haemangioblastoma-like clear cell stromal tumour, pulmonary, pathology

## Abstract

**Background:**

Hemangioblastoma-like clear cell stromal tumor (HLCCST) is a recently reported neoplasm of the lung. Only 13 cases have been reported in four recent studies. Because HLCCST is very rare, it has not been included in the 2021 WHO classification of lung tumors.

**Case Presentation:**

We report a case of HLCCST of the left lower lung in a 40-year-old female who was admitted to our hospital after pulmonary nodules were discovered. A plain chest CT scan showed a nodular high-density shadow measuring approximately 8 mm in diameter in the left lower lung. The lesion had clear borders, uneven internal density, and a low-density central vacuolar area. The left lower lung was partially resected by video-assisted thoracic surgery. Post-operative histopathologic diagnosis “hemangioblastoma-like clear cell stromal tumor” of the left lower lung.

**Conclusion:**

The HLCCST is an extremely rare tumor and needs long-term follow-up after operation. Clinically, it may be easily confused with other benign and malignant tumors of the lung, and diagnosis is solely determined by histopathologic examination. This case suggests that immunohistochemical CD34 can be a strong positive marker.

## Introduction

Hemangioblastoma is a tumor that typically occurs in the central nervous system, particularly in the cerebellum, and is largely related to Von Hippel-Lindau (VHL) syndrome (1). Previously, it was uncommon to find Hemangioblastoma outside the central nervous system, although it has been reported in the liver, kidney, pancreas, and other parenchymal organs (2). Primary pulmonary neoplasms are mostly epithelial in origin, with adenocarcinoma being the most common; primary mesenchymal tumors of the lung are considered unusual (3). Therefore, it is extremely rare to find a hemangioblastoma-like clear cell stromal tumor in the lung. To date, there are only 13 known cases reported in the literature. Herein, we report a case confirmed by post-operative histopathology and review the relevant literature to improve the understanding of surgeons and pathologists, to reduce misdiagnosis and incorrect treatment.

## Case Description

### Case Presentation

**FIGURE 1:**
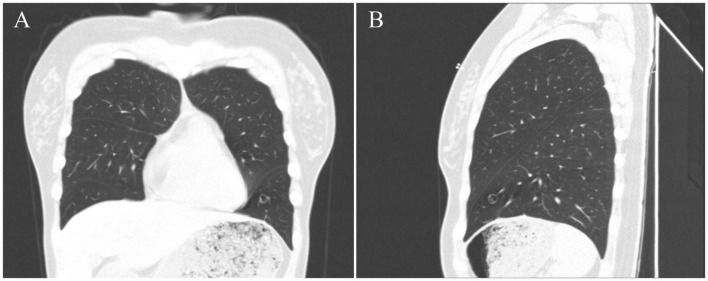
CT images of this case. CT images [**(A)**, coronal position] [**(B)**, sagittal position] show the tumor nodular high-density shadow in the lower lobe of the left lung, with a clear boundary, uneven internal density, and low-density vacuolar area in the center of the lesion.

The patient was a 40-year-old female who was admitted to our hospital after pulmonary nodules were detected incidentally, a year before presentation, in a chest CT. No cough, expectoration, chest tightness, shortness of breath, nausea, vomiting, low-grade fever, or night sweats were reported. On physical examination, her trachea was midline, and no deformities were noted in the thorax. Her breathing was stable and breath sounds were clear with no dry or wet rales; the activity of both the lungs was normal, and there was no obvious pleural friction. A plain chest CT scan showed a nodular high-density shadow, measuring approximately 8 mm in diameter, in the left lower lung. The lesion had clear borders, uneven internal density, and a low-density central vacuolar area (Figure 1). The left lower lung was partially resected by video-assisted thoracic surgery (VATS) was performed, and intra-operative frozen section pathological examination reported: “mesenchymal tumor of the left lower lung, the final result needs immunohistochemistry.” Post-operative histopathological diagnosis was a hemangioblastoma-like clear cell stromal tumor of the left lower lung. The patient was followed up regularly for 3 months after the surgery, with no recurrence or development of distant metastasis.

### Histopathology

The specimen submitted was from a wedge resection of the left lower lung lobe. The report of the gross specimen examination described the tumor as follows: postoperative gross specimen, left lower lung; wedge resection specimen: 7.9 × 6.3 × 4.1 cm, located 3.8 cm from the fault edge, with a peripheral gray-brown area of 0.9 × 0.8 cm seen in the lung tissue. The section was described as gray-white to gray-brown, with holes and unclear borders.

**FIGURE 2:**
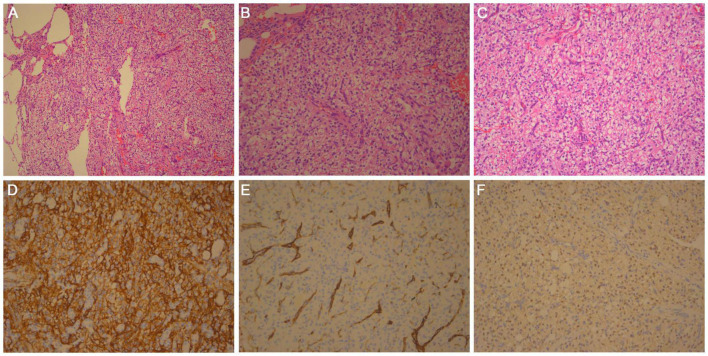
Histologic findings: hematoxylin and eosin-stained section of **(A)** × 100, **(B)** × 200, and **(C)** × 400 magnifications show that the tumor boundary is clear, composed of solid, slightly lobulated flake cells with consistent morphology. Scattered dilated vacuolar components can be seen in the tumor cells. **(D)** Tumor cells are positive for CD34. IHC × 200. **(E)** Tumor cells are negative for F8, and vessel positive. IHC × 200. **(F)** Tumor cells are weakly positive for TFE3. IHC × 200.

Microscopic analysis showed that the tumor was composed of solid, slightly lobulated flake cells with consistent morphology, and scattered dilated vacuolar components within the cells. Tumor cells were noted to be of medium size, with unclear borders, and a bright cytoplasm. The nucleus was deeply stained, oval to spindle-shaped. The nucleolus was not visible, and no necrosis or mitosis was noted (Figure 2). Immunohistochemistry (IHC) staining results revealed the expression of vimentin, Transcription factor E3 (TFE3) (weak), CD34 (strong), and Ki-67 (only 2% expression). The specimen was negative for HMB45, melan-A, S-100, Syn, CgA, CD56, napsin A, TTF-1, CK (AE1/AE3), PAX8, GFAP, desmin, MSA, inhibin-α, CD31, F8, DOG1, STAT6, ERG, ER, CAIX, and CD10 (Figure 2). Periodic acid-Schiff (PAS) staining was also negative. Fluorescent *in situ* hybridization (FISH) showed none Yes-associated protein 1 (*YAP1*)*-TFE3* gene fusions.

## Discussion

Hemangioblastoma are vascular neoplasms that typically occur in the central nervous system. However, they may be rarely found in the retroperitoneum, pelvic cavity, presacral region, kidney, maxillary sinus, and adrenal gland (4). In 2013, Falconieri et al. (5) reported the first two cases of pulmonary clear cell stromal tumors with previously unreported histological features similar to hemangioblastoma, which they named “hemangioblastoma-like clear cell stromal tumor of the lung”. The HLCCST is very uncommon, to the best of the authors’ knowledge, only 13 cases have been reported in four articles. Due to its rarity, it has not been included in the 2021 WHO classification of pulmonary mediastinal tumors. We reported a case of histopathologically confirmed HLCCST; compared to the previous literature, our case had distinctive IHC expression.

In the 13 cases discussed, the age range of patients was 29–77 years old. Among these, nine were women and four were men. The VHL syndrome, which is characterized by the presence of neoplasms affecting the central nervous system, kidneys, adrenal glands, pancreas, and reproductive organs (6), was not reported in any of the 13 patients. The clinical manifestations were non-specific: cough, dyspnea, fever, and hemoptysis with or without chest pain. Similarly, we report a 40-year-old female patient who was asymptomatic.

The gross examination of the specimen reported in the 13 cases revealed that the tumors were all non-encapsulated, but were clearly defined masses, with gray-yellow sections, focal bleeding, and with a maximum diameter of approximately 2–9.5 cm. Most tumors were found in the lung parenchyma, with a single case occurring in the bronchial lumen of the patient. Microscopically, the tumor was composed of solid flake cells with consistent morphology, slightly lobulated, with scattered dilated vacuolar components observed in the tumor cells. Tumor cells are of medium size, with unclear boundaries, and bright cytoplasm. The nucleus was deeply stained, oval to spindle-shaped, and the nucleolus was not visible. Vitreous degeneration was seen in the focal area, without necrosis and mitosis. Adipocytes could be seen in some cases.

The IHC staining in the previous reports showed that tumor cells expressed vimentin, and were positive for TFE3, but were negative for CK, EMA, TTF-1, P40, CGA, SYN, CD56, CD34, CD31, STAT6, desmin, calponin, SMA, GATA3, SOX-10, Bcl-2, S-100, HMB-45, etc. (7). The PAS staining was also negative. *YAP1-TFE3* gene fusions have recently been reported in 5 cases (8). The *YAP1-TFE3* fusion has only been described in a rare subset of epithelioid hemangioendothelioma (EHE), which was lacked the classic WW domain containing transcription regulator 1-Calmodulin binding transcription activator 1 (*WWTR1-CAMTA1*) fusion (9). In our case, IHC staining showed that tumor cells were positive for vimentin, strong expression of CD34, and weakly positive for TFE3; and FISH showed none *YAP1-TFE3* gene fusions. Expression of *TFE3* was not consistent with *YAP1-TFE3* gene fusions in HLCCST. Similar to most of the previously reported cases, in this case, the tumor expressed vimentin and TFE3. However, it also strongly expressed CD34, which only 1 of the 13 cases previously reported focally expressed CD34. The FISH results were also different compared to the previous reports.

Differential diagnosis based on histopathology includes carcinoid tumor, paraganglioma, perivascular epithelioid cell tumor (PEComa) (clear cell “sugar” tumor), and intrapulmonary solitary fibrous tumor. Because HLCCST is a diagnosis of exclusion, a wide panel of IHC stains is particularly important. Carcinoid tumors may express neuroendocrine markers (i.e., SYN, CGA, and CD56) and epithelial markers (i.e., CK). Paraganglioma can also express neuroendocrine markers (i.e., SYN, CGA, and CD56), and S-100 is expressed in the sustentacular cells. The PEComa may express myocyte-derived and melanocyte-derived markers, such as actin, desmin, HMB45, and melan-A. Intrapulmonary solitary fibrous tumors can express CD34 and STAT6. This case was positive for CD34, but negative for STAT6, therefore, a solitary fibrous tumor was excluded.

Finally, it is also important to distinguish the tumor from hemangioblastoma, which can specifically express S-100 and inhibin-α. In FISH analysis, *YAP1-TFE3* gene fusion can increase confidence in the diagnosis, but it is not a prerequisite (10).

At present, the HLCCST is considered to have a benign biological behavior. To our knowledge, none of the patients had recurrence or distant metastasis after complete resection. We believe that the first choice of treatment for HLCCST should be partial pneumonectomy *via* VATS and that regular postoperative follow-up is crucial.

## Conclusion

In summary, the HLCCST is an extremely rare tumor and needs long-term follow-up after operation. Clinically, it may be easily confused with other benign and malignant tumors of the lung, and diagnosis depends on histopathologic examination.

## Data Availability Statement

The original contributions presented in the study are included in the article/supplementary material, further inquiries can be directed to the corresponding author.

## Author Contributions

XZ and HW acquired the data. BH analyzed the histological images. XZ, HW, BH, and HJ prepared the manuscript. All authors contributed to the article and approved the submitted version.

## Conflict of Interest

The authors declare that the research was conducted in the absence of any commercial or financial relationships that could be construed as a potential conflict of interest.

## Publisher’s Note

All claims expressed in this article are solely those of the authors and do not necessarily represent those of their affiliated organizations, or those of the publisher, the editors and the reviewers. Any product that may be evaluated in this article, or claim that may be made by its manufacturer, is not guaranteed or endorsed by the publisher.

## Abbreviations

*YAP1*: Yes-associated protein 1
TFE3: Transcription factor E3
CT: Computed tomography
VATS: Video-assisted thoracic surgery
HLCCST: Hemangioblastoma-like clear cell stromal tumor
VHL: Von Hippel-Lindau
FISH: fluorescence *in situ* hybridization
IHC: Immunohistochemistry.
WWTR1: WW domain containing transcription regulator 1
CAMTA1: Calmodulin binding transcription activator 1
PEComa: Perivascular epithelioid cell tumor.
